# Supplementary material for: Toxicity of Metal Oxide Nanoparticles: Looking through the Lens of Toxicogenomics
Source: Int J Mol Sci. 2023 Dec 30;25(1):529. doi: 10.3390/ijms25010529 (PMC10779048; doi:10.3390/ijms25010529)
Supplement: Supplementary file 1 [file ijms-25-00529-s001.zip › Supplementary Document 1_ProofRevised.pdf]

1.1 Enriched canonical pathway in-depth overview:

Overall, the pathway responses seen across exposure conditions were largely compound specific, with metal chlorides and NPs inducing qualitatively similar responses, albeit with potency and enrichment differences.

i. ZnO NPs, MPs, $ZnCl_2$:

When compared based on the amount of constituent Zn in the exposure media, ZnO MPs present the strongest response at 24 and 48 h, but the weakest response at 2 h (Supplementary File 1). For both NPs and MPs, the 5 μg/mL concentration was the only one where significantly enriched pathways were seen at any timepoint.

At the early timepoint of 2 h, 5 μg/mL ZnO NPs enriched 98 pathways, many related to cellular stress and death (ex. 'Unfolded protein response', 'NRF2-mediated Oxidative Stress Response', 'Ferroptosis Signaling Pathway', 'HIF1α Signaling'), immune signaling and disease (ex 'IL-10 Signaling', 'IL-6 Signaling', 'Acute Phase Response Signaling'), in addition to multiple metabolic and signaling related responses. For ZnO MPs at 5 μg/mL, the low-fold-change set featured 2 pathways, the 'Aldosterone Signaling in Epithelial Cells', and 'Protein Ubiquitination Pathway', while the high-fold-change set included 9 pathways relating mainly to cellular stress ('HIF1α Signaling', 'NRF2-mediated Oxidative Stress Response', 'Protein Ubiquitination Pathway') and signaling, in addition to the 'Phagosome Formation' pathway. With respect to $ZnCl_2$, 9 and 101 pathways were enriched at 5.1 and 8.5 μg/mL at 2 h. Notably in common between the two concentrations were pathways relating to disease (ex. 'HER-2 Signaling in Breast Cancer', 'Role of PKR in Interferon Induction and Antiviral Response', 'Huntington's Disease Signaling'), in addition to the 'HIF1α Signaling' pathway. Uniquely enriched at the 8.5 μg/mL concentration were 93 pathways, many relating to cellular stress (ex. 'Unfolded protein response', 'NRF2-mediated Oxidative Stress Response', 'Ferroptosis Signaling Pathway'), immune response, (ex. 'TNFR2 Signaling', 'IL-10 Signaling', 'IL-7 Signaling Pathway'), wound healing and disease (ex. 'Wound Healing Signaling Pathway', 'Pulmonary Fibrosis Idiopathic Signaling Pathway', 'Molecular Mechanisms of Cancer') and general signaling.

By 24 h, response to ZnO NPs increased, with 149 significantly enriched pathways at 5 μg/mL. Many of the same responses from the 2 h timepoint were still present, including cellular stress and cell death related processes (ex. 'Unfolded protein response', 'NRF2-mediated Oxidative Stress Response', 'Ferroptosis Signaling Pathway', 'HIF1α Signaling'), and immune response and disease pathways (ex. 'Acute Phase Response Signaling', 'IL-10 Signaling', 'IL-6 Signaling'). In addition, enrichment of new pathways related to vesicular trafficking was seen ('CLEAR Signaling Pathway', 'Autophagy', 'Caveolar-mediated Endocytosis Signaling', 'Clathrin-mediated Endocytosis Signaling', 'Phagosome Maturation'). The 5 μg/mL ZnO MPs at this timepoint featured 228 unique pathways between both low and high-fold-change sets, with 89 pathways in the low group, 170 pathways in the high group, and 31 pathways shared between the two sets. Response to ZnO MPs at this timepoint was more in line with responses seen for ZnO NPs and $ZnCl_2$ than at 2 h, including pathways relating to cell stress and death (ex. 'NRF2-mediated Oxidative Stress Response', 'Unfolded protein response', 'Ferroptosis Signaling Pathway'), immune response and disease (ex. 'Acute Phase Response Signaling'), vesicular trafficking ('CLEAR Signaling Pathway', 'Caveolar-mediated Endocytosis Signaling', 'Autophagy') and general signaling and metabolic pathways. Finally for $ZnCl_2$ at 24 h, 25 and

177 pathways were enriched at 5.1 and 8.5 μg/mL with high similarity to 5 μg/mL of NPs and MPs. Of those pathways 13 were commonly enriched, mainly relating to immune response (ex. 'Pathogen Induced Cytokine Storm Signaling Pathway', 'Toll-like Receptor Signaling') and metabolism (ex. 'Xenobiotic Metabolism Signaling'). Unique to the high concentration group were an additional 164 pathways related to similar processes seen for NPs and MPs, including cell stress and death (ex. 'Unfolded protein response', 'NRF2-mediated Oxidative Stress Response', 'Ferroptosis Signaling Pathway'), immune response and disease (ex. 'TGF-β Signaling', 'IL-8 Signaling', 'Chemokine Signaling'), vesicular trafficking ('CLEAR Signaling Pathway', 'Autophagy', 'Caveolar-mediated Endocytosis Signaling', 'Phagosome Maturation', 'Clathrin-mediated Endocytosis Signaling'), as well as signaling and metabolism.

For the latest timepoint of 48 h, a reduced number of pathways was seen with each form of Zn. For 5 μg/mL ZnO NPs, 95 pathways were significantly enriched, with noted induction of DNA repair and damage response pathways (ex. 'Role of BRCA1 in DNA Damage Response', 'Mismatch Repair in Eukaryotes', 'NER (Nucleotide Excision Repair, Enhanced Pathway)', 'ATM Signaling'), cell stress and death responses (ex. 'p53 Signaling', 'Ferroptosis Signaling Pathway', 'HIF1α Signaling', 'Senescence Pathway, 'NRF2-mediated Oxidative Stress Response', 'Unfolded protein response'), immune signaling and disease (ex. 'Pathogen Induced Cytokine Storm Signaling Pathway', 'Interferon Signaling', 'Pancreatic Adenocarcinoma Signaling'), however all pathways relating to vesicular trafficking seen at 24 h were no longer enriched at 48 h. With respect to 5 μg/mL ZnO MPs at 48 h, 199 unique pathways were enriched across both high and low fold change groups, with 89 pathways in the low-fold-change group, 133 pathways in the high-fold-change group, and 23 pathways shared between the two. Similar responses to 5 μg/mL ZnO NPs were seen, including induction of DNA damage and repair pathways (ex. 'Role of BRCA1 in DNA Damage Response', 'NER (Nucleotide Excision Repair, Enhanced Pathway)', 'Mismatch Repair in Eukaryotes'), cell stress and death signaling (ex. 'p53 Signaling', 'Ferroptosis Signaling Pathway', 'HIF1α Signaling', 'Unfolded protein response', 'NRF2-mediated Oxidative Stress Response'), and immune response and disease (ex. 'Atherosclerosis Signaling', 'Interferon Signaling', 'Pathogen Induced Cytokine Storm Signaling Pathway'). In addition, the 5 μg/mL low-fold-change set notably enriched vesicular trafficking pathways no longer seen in ZnO NPs (ex. 'Caveolar-mediated Endocytosis Signaling', 'Autophagy', 'CLEAR Signaling Pathway). Finally, $ZnCl_2$ at 48 h lead to the enrichment of 36 and 146 pathways at 5.1 and 8.5 μg/mL respectively. Between the two concentrations 28 pathways were shared, which included DNA damage and repair processes (ex. 'DNA damage-induced 14-3-3σ Signaling', 'ATM Signaling', 'Role of BRCA1 in DNA Damage Response'), cell cycle responses (ex. 'Kinetochore Metaphase Signaling Pathway', 'Mitotic Roles of Polo-Like Kinase', 'Cyclins and Cell Cycle Regulation'), immune signaling and disease, (ex. 'Acute Phase Response Signaling', 'IL-10 Signaling', 'Pulmonary Fibrosis Idiopathic Signaling Pathway'), and cellular stress ('p53 Signaling', 'Senescence Pathway'). Unique to 8.5 μg/mL at 48 h were 118 pathways implicated in DNA repair (ex. 'BER (Base Excision Repair) Pathway', 'NER (Nucleotide Excision Repair, Enhanced Pathway)', 'Mismatch Repair in Eukaryotes), cell stress and death (ex. 'Ferroptosis Signaling Pathway', 'HIF1α Signaling', 'NRF2-mediated Oxidative Stress Response', 'Unfolded protein response'), immune response and disease (ex. 'Interferon

Signaling', 'IL-7 Signaling Pathway', 'TGF-β Signaling', 'Molecular Mechanisms of Cancer'), as well as numerous pathways implicated in signaling and metabolic processes.

ii. *CuO NPs, MPs, $CuCl_2$:*

The pathway response to CuO NPs, MPs, and $CuCl_2$ has been described in detail in Boyadzhiev et al., 2021. Analysis of the published data with the most recent version of IPA used in this study resulted in much the same responses with some notable differences (Supplementary File 2).

At the 2 h timepoint only 25 μg/mL CuO MPs featured significantly two enriched pathways, with 'Mitotic Roles of Polo-like Kinase' and 'Protein Ubiquitination Pathway' being the only perturbed responses. In the previously conducted IPA analysis, CuO MPs were not examined in IPA, but using GO enrichment. Using the GO ontologies, it could be seen that 'regulation of meiotic cell cycle' [GO:0051445] and 'anaphase-promoting complex-dependent catabolic process' [GO:0031145], as well as the cellular components 'nuclear ubiquitin ligase complex' [GO:0000152] and 'anaphase-promoting complex' [GO:0005680] were significantly enriched in the down regulated gene list [34].

At the 24 h timepoint, only CuO NPs presented enriched pathways with 10, 67, and 89 pathways at 5, 10, and 25 μg/mL. At this timepoint, 5, 10, and 25 μg/mL concentrations commonly induced disturbances in 6 pathways relating to vesicular trafficking ('CLEAR Signaling Pathway', 'Phagosome Maturation') and immune function ('IL-10 Signaling', 'Role of MAPK Signaling in Promoting the Pathogenesis of Influenza'), as well as the iron-mediated cell death pathway 'Ferroptosis Signaling Pathway'. At higher exposure concentrations of 10 and 25 μg/mL 30 pathways were commonly enriched, notably including numerous cell stress pathways (ex. 'HIF1a Signaling', 'NRF2-mediated Oxidative Stress Response', 'Unfolded protein response', 'BAG2 Signaling Pathway'), the vesicular trafficking pathway 'Autophagy', and multiple disease and metabolism related responses. The highest concentration of 25 μg/mL uniquely enriched 53 pathways, including DNA damage and repair responses (ex. 'Role of BRCA1 in DNA Damage Response', 'Mismatch Repair in Eukaryotes'), cell cycle and cancer responses (ex. 'Role of CHK Proteins in Cell Cycle Checkpoint Control', 'Cell Cycle Control of Chromosomal Replication', 'Molecular Mechanisms of Cancer'), and cellular stress and death signaling (ex. 'Immunogenic Cell Death Signaling Pathway', 'p53 Signaling', 'Death Receptor Signaling'). These responses are in line with the previously conducted IPA enrichment with 1, 20, and -1 extra pathways in comparison [34], and the notable enrichment of the 'CLEAR Signaling Pathway' which was not present previously. In addition, the 'BAG2 Signaling Pathway' was not enriched for 5 μg/mL CuO NPs at 24 h due to increases in the total gene count of the pathway (80 genes in IPA content version 81348237 vs 40 genes in IPA content version 48207413).

At the last timepoint of 48 h exposure to 5 and 10 μg/mL CuO NPs resulted in the strongest response with 96 and 170 enriched pathways respectively, whereas for 25 μg/mL no usable RNA was able to be extracted from exposed cells and was therefore not analyzed for transcriptional response (Boyadzhiev et al., 2021). In common to both 5 and 10 μg/mL CuO NP exposures were 74 pathways, including perturbed cell stress signaling responses (ex. 'NRF2-mediated Oxidative Stress Response', 'BAG2 Signaling Pathway', 'Unfolded protein response'), DNA damage and repair pathways (ex. 'GADD45 Signaling', 'ATM Signaling', 'Mismatch

Repair in Eukaryotes'), and pathways related to vesicular trafficking and turnover (ex. 'Autophagy', 'CLEAR Signaling Pathway', 'Caveolar-mediated Endocytosis Signaling'), in addition to multiple disease and cancer related pathways and metabolic and signaling responses. Unique to the 10 μg/mL concentration were 96 additional pathways related to cell stress (ex. 'Senescence Pathway', 'Necroptosis Signaling Pathway'), DNA repair (ex. 'BER (Base Excision Repair) Pathway', 'NER (Nucleotide Excision Repair, Enhanced Pathway)'), immune responses (ex. 'IL-10 Signaling', 'TGF-β Signaling', 'Acute Phase Response Signaling'), carcinogenesis (ex. 'Small Cell Lung Cancer Signaling', 'Renal Cell Carcinoma Signaling'), and general signaling and metabolism. Finally, with respect to 7 μg/mL $CuCl_2$, 51 pathways were enriched at 48 h including multiple stress pathways (ex. 'NRF2-mediated Oxidative Stress Response', 'Senescence Pathway', 'HIF1α Signaling'), as well as pathways relating to DNA damage response (ex. 'Cell Cycle: G2/M DNA Damage Checkpoint Regulation', 'ATM Signaling'), detoxification (ex. 'Glutathione-mediated Detoxification', 'Superoxide Radicals Degradation'), and cell cycle progression (ex. 'Cyclins and Cell Cycle Regulation', 'Role of CHK Proteins in Cell Cycle Checkpoint Control'). In comparison with the IPA analysis conducted in Boyadzhiev et al 2021 [34], 16 and 43 additional pathways are seen for CuO NPs, and 14 additional pathways are seen for $CuCl_2$. Much the same response is seen for 5 and 10 μg/mL CuO NPs, with cell stress, DNA damage, and cell death pathways making up the majority of the highly enriched pathways. Similarly, the most affected processes involved in $CuCl_2$ response at 48 h are akin to the previous IPA enrichment [34], which include oxidative stress, DNA damage, cell cycle progression, and cellular detoxification.

iii. NiO NPs, MPs, $NiCl_2$:

For Ni compounds, NiO NPs and $NiCl_2$ presented much the same response profile, at the same timepoints of assessment, albeit at different concentrations of Ni whereas NiO MPs generally presented a similar but muted response (Supplementary Data File 3).

At 2 h, no pathway enrichment was seen for any Ni compound.

At 24 h, NiO NPs featured 4, 46, and 80 enriched pathways at 10, 25, and 50 μg/mL. All pathways enriched at 10 μg/mL were commonly enriched at 25 and 50 μg/mL, with 'Glycolysis' 'Gluconeogenesis I', and 'Sirtuin Signaling Pathway' notably enriched. Higher concentrations of 25 and 50 μg/mL commonly enriched 36 pathways, including multiple DNA damage response and repair pathways (ex. 'NER (Nucleotide Excision Repair, Enhanced Pathway)', 'Mismatch Repair in Eukaryotes', 'Role of BRCA1 in DNA Damage Response'), and cell stress pathways ('HIF1a Signaling', 'Senescence Pathway', 'Aryl Hydrocarbon Receptor Signaling'). The highest concentration of 50 μg/mL uniquely enriched 40 additional pathways at this timepoint, including additional cell stress related responses (ex. 'NRF2-mediated Oxidative Stress Response', 'Endoplasmic Reticulum Stress Pathway', 'Unfolded protein response'), and pathways related to vesicular trafficking ('CLEAR Signaling Pathway', 'Phagosome Maturation', 'Autophagy'). For NiO MPs at 24 h, 9 and 50 pathways were significantly enriched at 25 and 50 μg/mL. Common between the two concentrations were 8 pathways, including the 'HIF1a Signaling', 'Sirtuin Signaling Pathway', 'Glycolysis I', and 'Gluconeogenesis I' pathways implicated in hypoxia. The higher concentration of 50 μg/mL further uniquely enriched 42 pathways, 23 of which relate to disease and immune response (ex. 'Interferon Signaling', 'IL-33 Signaling Pathway', 'Neuroinflammation Signaling Pathway'), 7 associated with cell stress /

aberrant wound healing (ex. 'Ferroptosis Signaling Pathway', 'NRF2-mediated Oxidative Stress Response', 'Role of Tissue Factor in Cancer'), and the rest comprising metabolic and signaling pathways. With respect to specific pathways related to $NiCl_2$ at 24 h, 58 and 93 pathways were enriched at 40 and 80 μg/mL. Of these pathways, 49 were commonly enriched between the 40 and 80 μg/mL concentrations, including 'HIF1a Signaling', 'Glycolysis I', and 'Gluconeogenesis I' pathways noted for NiO NPs and MPs. The remaining pathways were comprised of DNA damage and repair responses (ex. 'Role of BRCA1 in DNA Damage Response', 'NER (Nucleotide Excision Repair, Enhanced Pathway)', 'GADD45 Signaling'), cell stress and disease (ex. 'p53 Signaling', 'Senescence Pathway', 'Pulmonary Fibrosis Idiopathic Signaling Pathway'), vesicular trafficking processes ('CLEAR Signaling Pathway', 'Autophagy', 'Caveolar-mediated Endocytosis Signaling'), and pathways related to cell cycle control, carcinogenesis, metabolism and signaling. Unique to the 80 μg/mL concentration at 24 h were 44 pathways mainly involved in cell stress, immune signaling, and metabolism, with 'Oxidative Phosphorylation', 'Mitochondrial Dysfunction', and 'NRF2-mediated Oxidative Stress Response' notably enriched.

By 48 h, the concentration-dependent response to NiO NPs increased, with 10, 90, and 147 significantly enriched pathways for 10, 25, 50 μg/mL exposure groups. Common to all concentrations of NiO NPs at this timepoint were 6 pathways, most of which relate to hypoxia ('Glycolysis I', 'Gluconeogenesis I', 'HIF1α Signaling', 'Sirtuin Signaling Pathway'). At higher concentrations of 25 and 50 μg/mL, 53 pathways were commonly enriched including multiple immune related pathways (ex. 'Acute Phase Response Signaling', 'IL-10 Signaling', 'Pathogen Induced Cytokine Storm Signaling Pathway'), some cell stress pathways ('Senescence Pathway', 'Oxidative Phosphorylation'), and pathways related to DNA damage response ('GADD45 Signaling', 'Cell Cycle: G2/M DNA Damage Checkpoint Regulation'). Unique to 50 μg/mL NiO NPs at 48 h timepoint were 88 additional pathways with notable cell stress responses (ex. 'Mitochondrial Dysfunction', 'NRF2-mediated Oxidative Stress Response', 'Unfolded protein response', 'Ferroptosis Signaling Pathway'), pathways relating to vesicular trafficking ('Autophagy', 'Caveolar-mediated Endocytosis Signaling', 'CLEAR Signaling Pathway'), additional immune related processes (ex. 'Interferon Signaling', 'IL-4 Signaling', 'Chemokine Signaling'), and multiple metabolic and signaling pathways. In terms of NiO MPs at 48 h, 17 and 37 enriched pathways were observed at 25 and 50 μg/mL respectively. Of those pathways, 9 were commonly enriched between both exposure groups, including hypoxia signaling pathways ('Glycolysis I', 'HIF1α Signaling', 'Gluconeogenesis I', 'Sirtuin Signaling Pathway'), and multiple pathways related to immune response and disease (ex. 'Atherosclerosis Signaling', 'Hepatic Fibrosis / Hepatic Stellate Cell Activation'). The high concentration of 50 μg/mL uniquely enriched 28 pathways of which 11 related to immune response and disease (ex. 'Toll-like Receptor Signaling', 'Hepatic Fibrosis Signaling Pathway') and 2 more related to metal homeostasis ('Inhibition of Matrix Metalloproteases', 'Ferroptosis Signaling Pathway'), with the rest being signaling and metabolic in nature. Finally with respect to $NiCl_2$ exposed cells at 48 h, 40 and 80 μg/mL concentrations enriched 81 and 153 pathways respectively, with 57 pathways in common between the two concentrations. These pathways were implicated in hypoxia signaling ('HIF1a Signaling', 'Glycolysis I', 'Gluconeogenesis I', 'Sirtuin Signaling Pathway'), immune and disease (ex. 'Interferon Signaling', 'Pulmonary Fibrosis Idiopathic Signaling Pathway', 'IL-10 Signaling'), and cell stress processes (ex. 'Mitochondrial dysfunction', 'p53

Signaling', 'Ferroptosis Signaling'), in addition to multiple metabolic and general signaling pathways. At the high $NiCl_2$ concentration of 80 μg/mL, 96 pathways were uniquely enriched including notable cell stress responses ('Unfolded protein response', 'NRF2-mediated Oxidative Stress Response'), vesicular trafficking pathways ('Autophagy', 'CLEAR Signaling Pathway', 'Caveolar-mediated Endocytosis Signaling'), immune processes (ex. 'Acute Phase Response Signaling', 'TGF-β Signaling', 'Pulmonary Healing Signaling Pathway'), and numerous signaling and metabolic pathways.

iv. Al_2O_3 NPs, MPs, $AlCl_3$:

With respect to Al exposures, Al_2O_3 NPs presented the weakest response of the MONPs tested (Supplementary Data File 4). At equivalent concentrations of constituent metal, $AlCl_3$ induced the most pronounced pathway response out of the three materials tested.

At 2 h, no significantly enriched pathways were seen for any Al material.

By the 24 h timepoint, Al_2O_3 NPs featured 3 enriched pathways, which consisted of the 'LPS/IL-1 Mediated Inhibition of RXR Function', 'Regulation Of The Epithelial Mesenchymal Transition By Growth Factors Pathway', and 'NRF2-mediated Oxidative Stress Response'. With respect to Al_2O_3 MPs, they featured their strongest response at 24 h, with 71 enriched pathways at 50 μg/mL. These pathways mainly related to immune responses (ex 'Toll-like Receptor Signaling', 'Th2 Pathway', 'Acute Phase Response Signaling') and metabolism and general signaling, with a few implicated in wound healing and the cytoskeleton ('Wound Healing Signaling Pathway', 'Actin Cytoskeleton Signaling'), and vesicular trafficking ('Phagosome Formation', 'Caveolar-mediated Endocytosis Signaling'). Notably, there was a lack of cell stress signaling, with only the 'NRF2-mediated Oxidative Stress Response' showing significant enrichment. For $AlCl_3$ at 24 h, 1 and 6 pathways were significantly enriched at 47 and 118 μg/mL. The only pathway at 47 μg/mL was 'Glycolysis I', while the higher concentration of 118 μg/mL featured immune signaling (ex. 'Atherosclerosis Signaling', 'IL-17 Signaling'), as well as notable enrichment of the 'HIF1a Signaling' pathway.

At the latest timepoint of 48 h, Al_2O_3 NPs featured a concentration-dependent pathway response with 36 and 46 enriched pathways at 25 and 50 μg/mL respectively. Common to both concentrations at this timepoint were 26 pathways, including the 'HIF1a Signaling' pathway, as well as numerous pathways related to immune response and disease (ex. 'IL-6 Signaling', 'Acute Phase Response Signaling', 'Osteoarthritis Pathway') and aberrant wound healing (ex 'Pulmonary Healing Signaling Pathway', 'Pulmonary Fibrosis Idiopathic Signaling Pathway'). Unique to 50 μg/mL at this timepoint were 20 additional pathways, including the 'GADD45 Signaling' stress pathway and the 'Caveolar-mediated Endocytosis Signaling' internalization pathway, with the remaining pathways implicated in immune responses (ex. 'Toll-like Receptor Signaling', 'Role of IL-17A in Arthritis'), aberrant wound healing (ex. 'Regulation of the Epithelial-Mesenchymal Transition Pathway', 'Wound Healing Signaling Pathway', 'Bladder Cancer Signaling'), and general signaling. With respect to Al_2O_3 MPs at this timepoint, 14 pathways were enriched at 50 μg/mL representing a diverse range of functions including cell stress responses ('p53 Signaling', 'GADD45 Signaling'), immune responses ('LPS/IL-1 Mediated Inhibition of RXR Function'), and numerous cell signaling pathways. Finally with respect to 47 and 118 μg/mL $AlCl_3$, 45 and 60 pathways were significantly enriched. Commonly enriched between the two concentrations were 29 pathways mainly related to aberrant wound healing

and disease (ex. 'Pulmonary Fibrosis Idiopathic Signaling Pathway', 'Wound Healing Signaling Pathway', 'Hepatic Fibrosis Signaling Pathway'), as well as the 'Clathrin-mediated Endocytosis Signaling' pathway. Uniquely enriched at 118 µg/mL at 48 h were 31 additional pathways, including immune related responses (ex. 'Acute Phase Response Signaling', 'IL-17A Signaling in Fibroblasts'), pathways implicated in metal ion homeostasis ('Iron homeostasis signaling pathway', 'Ferroptosis Signaling Pathway', 'Inhibition of Matrix Metalloproteases'), and multiple metabolic and signaling responses.

v. TiO_2 NPs, MPs:

Transcriptional response to insoluble TiO_2 was much more pronounced in the case of TiO_2 NPs, as compared to TiO_2 MPs, (Supplementary Data File 5).

At the earliest timepoint of 2 h, TiO_2 NPs induced mild concentration dependent pathway enrichment with 1, 2, and 5 enriched pathways at 25, 50, and 100 µg/mL concentrations. All concentrations significantly enriched the 'Human Embryonic Stem Cell Pluripotency' pathway, while the 50 and 100 µg/mL concentrations also commonly enriched the 'Mouse Embryonic Stem Cell Pluripotency' pathway. Unique to 100 µg/mL were 2 additional pathways related to diseases, and the calcium signaling pathway 'S100 Family Signaling Pathway'. No significant pathway enrichment was seen for TiO_2 MPs at this timepoint.

By 24 h, concentration dependent enrichment was seen for both NPs and MPs. The response to TiO_2 NPs was more pronounced with 33, 66, and 99 enriched pathways at 25, 50, and 100 µg/mL concentrations. For TiO_2 NPs at this timepoint, 21 pathways were commonly shared between all concentrations including DNA repair pathways ('NER (Nucleotide Excision Repair, Enhanced Pathway)', 'Mismatch Repair in Eukaryotes'), vesicular trafficking responses ('CLEAR Signaling Pathway', 'Phagosome Maturation'), metal ion homeostasis related responses ('Iron homeostasis signaling pathway', 'Ferroptosis Signaling Pathway'), as well as general disease type and metabolic pathways. The two higher TiO_2 NP concentrations (50 & 100 µg/mL) commonly enriched 27 additional pathways, mainly related to DNA repair and damage response (ex. 'Role of BRCA1 in DNA Damage Response', 'GADD45 Signaling', 'BER (Base Excision Repair) Pathway'), and immune signaling (ex. 'IL-10 Signaling', 'IL-17A Signaling in Fibroblasts'), as well as both the 'Autophagy' and 'Senescence Pathway' processes. Unique to the 100 µg/mL exposure group were 50 further pathways implicated in DNA damage responses (ex. 'ATM Signaling', 'Nucleotide Excision Repair Pathway', 'Cell Cycle: G2/M DNA Damage Checkpoint Regulation), cell cycle control, (ex. 'Cyclins and Cell Cycle Regulation', 'Mitotic Roles of Polo-Like Kinase', 'Cell Cycle: G1/S Checkpoint Regulation'), vesicular trafficking ('Caveolar-mediated Endocytosis Signaling', 'Virus Entry via Endocytic Pathways'), as well as the 'p53 Signaling' pathway, and additional metabolic and signaling responses. When examining TiO_2 MPs at 24 h, it can be seen that 6 and 27 pathways were enriched at 50 and 100 µg/mL. All pathways enriched at 50 µg/mL were commonly enriched at 100 µg/mL, which includes the 'CLEAR Signaling Pathway' and 5 pathways related to general immune functions (ex. 'Granulocyte Adhesion and Diapedesis'). Unique to the 100 µg/mL concentration of TiO_2 MPs were 21 pathways related to immune functions (ex 'Acute Phase Response Signaling', 'IL-17 Signaling), vesicular trafficking ('Autophagy', 'Phagosome Maturation'), cellular stress (ex. 'NRF2-mediated Oxidative Stress Response', 'HIF1α Signaling'), metal ion homeostasis ('Iron

homeostasis signaling pathway', 'Inhibition of Matrix Metalloproteases'), and metabolic and signaling responses.

By the final timepoint of 48 h, both TiO_2 NPs and MPs featured concentration dependent pathway enrichment. With respect to TiO_2 NPs at this timepoint, 101, 162, and 262 pathways were significantly enriched at 25, 50, and 100 μg/mL concentrations respectively. Of these pathways, 84 pathways were commonly enriched across all concentrations, including multiple pathways implicated in aberrant wound healing and transformation (ex. 'Pulmonary Fibrosis Idiopathic Signaling Pathway', 'Regulation of the Epithelial-Mesenchymal Transition Pathway'), immune signaling and disease (ex. 'Acute Phase Response Signaling', 'Osteoarthritis Pathway'), vesicular trafficking ('Caveolar-mediated Endocytosis Signaling', 'Clathrin-mediated Endocytosis Signaling'), multiple signaling pathways, and the 'HIF1a Signaling' pathway. For the top two TiO_2 NP concentrations of 50 and 100 μg/mL, another 56 pathways were commonly enriched relating to cellular stress (ex. 'NRF2-mediated Oxidative Stress Response', 'BAG2 Signaling Pathway', 'GADD45 Signaling'), particle uptake and vesicular trafficking ('Virus Entry via Endocytic Pathways', 'CLEAR Signaling Pathway'), immune type responses (ex. 'IL-15 Production', IL-8 Signaling', 'IL-17 Signaling'), in addition to multiple metabolic, signaling and cancer related pathways. Unique to the 100 μg/mL concentration of TiO_2 NPs were 119 pathways, mainly implicated in cellular stress (ex. 'Apoptosis Signaling', 'Unfolded protein response', 'Autophagy'), immune responses (ex. 'IL-1 Signaling', 'Chemokine Signaling', 'LPS-stimulated MAPK Signaling'), and general signaling and metabolism. Finally, with respect to TiO_2 MPs at this timepoint, 14 and 40 pathways were noted for 50 and 100 μg/mL concentrations respectively. The two concentrations feature 11 common pathways almost all of which relate to immune signaling (ex. 'Acute Phase Response Signaling', 'Atherosclerosis Signaling', 'Agranulocyte Adhesion and Diapedesis'). Unique to the 100 μg/mL TiO_2 MP exposure group were 29 enriched pathways, including numerous additional immune signaling and disease pathways (ex. 'Osteoarthritis Pathway', 'Complement System', 'IL-13 Signaling Pathway'), cancer and aberrant wound healing responses (ex. 'Colorectal Cancer Metastasis Signaling', 'Pulmonary Fibrosis Idiopathic Signaling Pathway', 'Molecular Mechanisms of Cancer'), as well as two pathways implicated in metal ion homeostasis ('Iron homeostasis signaling pathway', 'Inhibition of Matrix Metalloproteases').
