# Supplementary material for: Toxicity of Metal Oxide Nanoparticles: Looking through the Lens of Toxicogenomics
Source: Int J Mol Sci. 2023 Dec 30;25(1):529. doi: 10.3390/ijms25010529 (PMC10779048; doi:10.3390/ijms25010529)
Supplement: Supplementary file 1 [file ijms-25-00529-s001.zip › Supplementary Figures_ProofRevisedFinal.pdf]

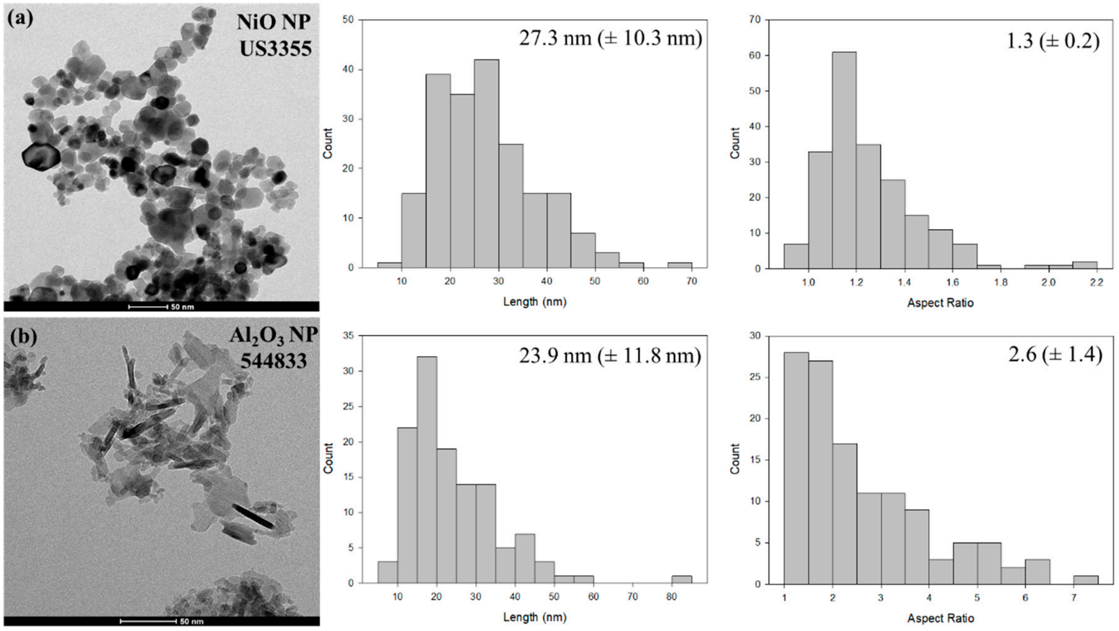

Figure S1. Representative TEM images of (a) NiO NPs and (b) Al_2O_3 NPs used in this study, along with the average length and aspect ratio based on TEM sizing (+/- standard deviation). Scale bar: 50 nm.

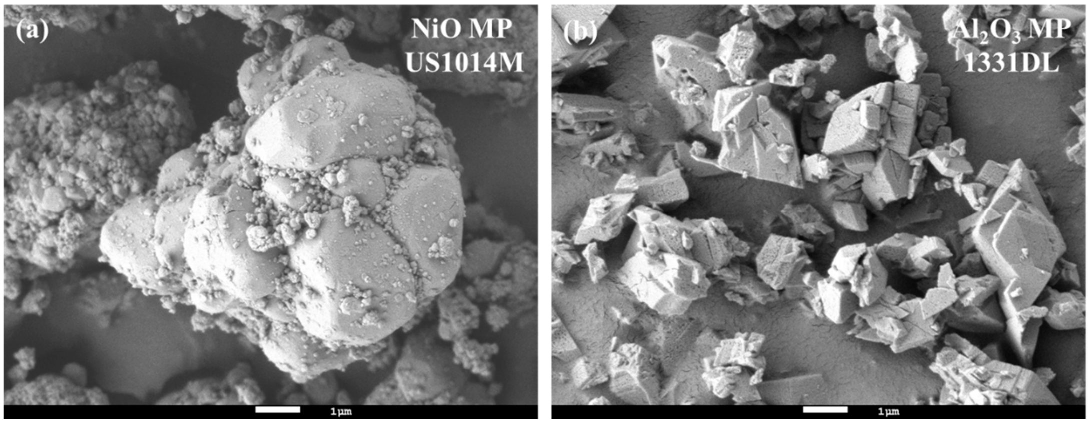

Figure S2. Representative SEM images of (a) NiO NPs and (b) Al_2O_3 MPs. Scale bar: 1 μm.

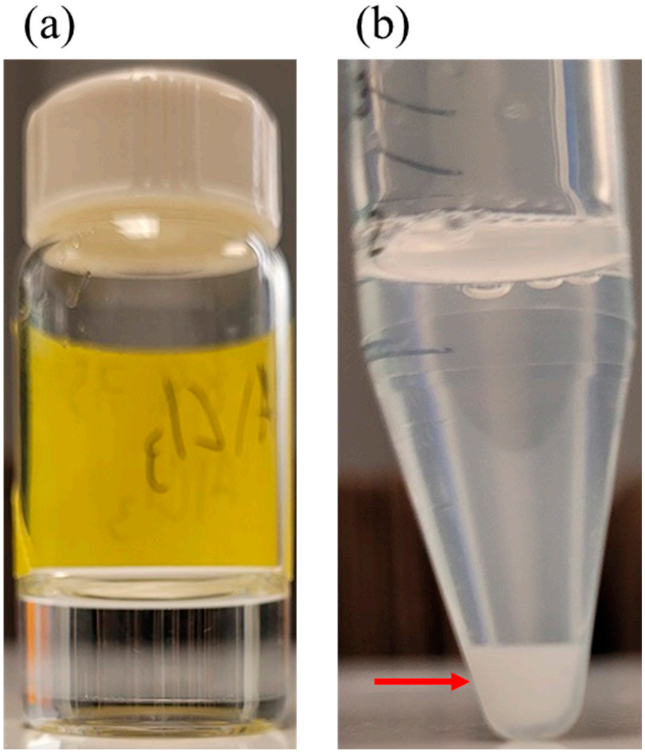

Figure S3. $AlCl_3$ dissolved in (a) ultrapure water or (b) in cell culture medium with serum. Red arrow: white precipitate formed when $AlCl_3$ dissolved in water was added to medium. Centrifugation was used to collect precipitate at the bottom of the tube.

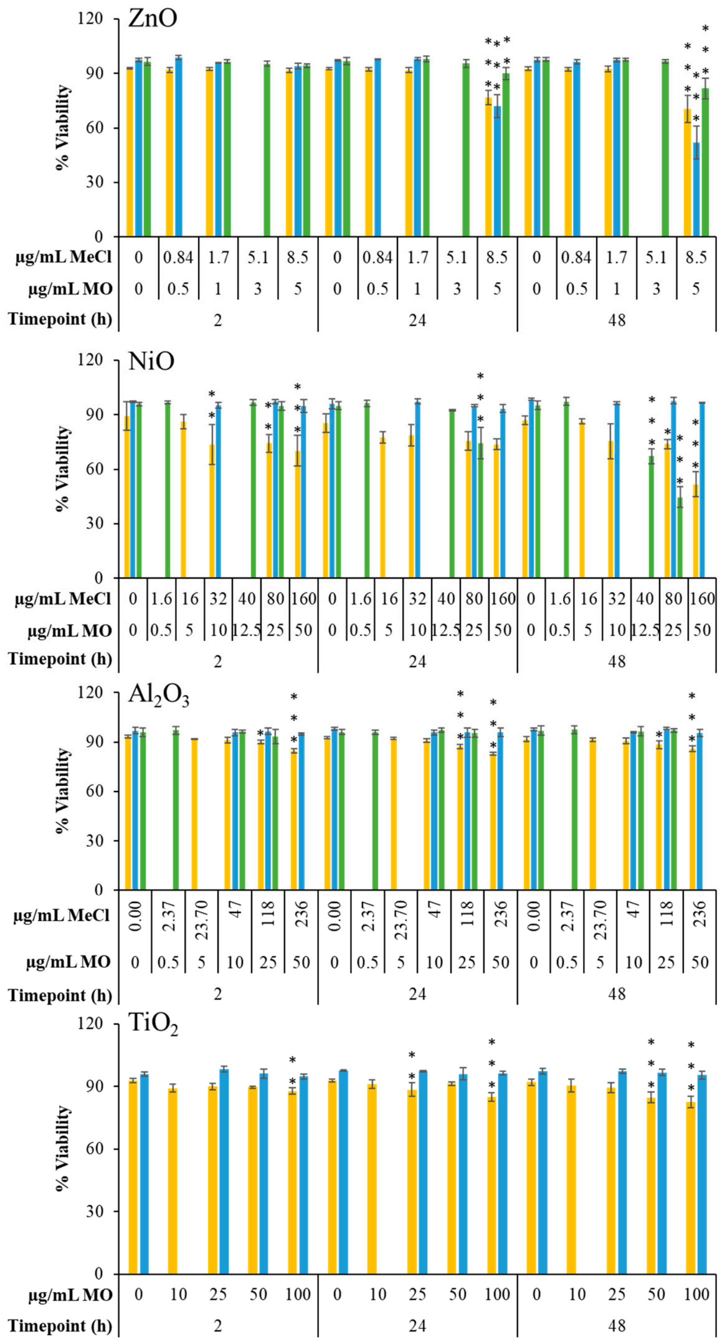

Figure S4. % Viability of FE1 cells following 2, 24, and 48 h of exposure to MONPs, MOMPs, and metal chlorides compared to time-matched medium controls. Error bars indicate standard deviation (n = 3-4). Graphs were labelled based on the type of metal oxide. Yellow: MONPs. Blue: MOMPs. Green: metal chloride salts. MO: metal oxide. MeCl: metal chloride. Statistical significance against time-matched medium controls were determined through a 2-way ANOVA with a Dunnett's post-hoc. *: $p < 0.05$. **: $p < 0.01$. ***: $p < 0.001$.

Table S1. Percent dissolution of MONPs and MOMPs used in this study after 0 – 48 H of incubation within DMEM + 2 % FBS cell culture medium. (-): not assessed.

Metal Oxide	Size	Timepoint (h)	10 µg/mL	100 µg/mL
ZnO[a]	NP	0	94 %	14.6 %
		2	(-)	(-)
		24	96.1 %	18.5 %
		48	94.5 %	19.3 %
	MP	0	(-)	12.5 %
		2	(-)	(-)
		24	(-)	11.7 %
		48	(-)	11.8 %
CuO[b]	NP	0	3.4 %	4.2 %
		2	5 %	16.5 %
		24	11.8 %	44.5 %
		48	12.6 %	51.6 %
	MP	0	(-)	0.24 %
		2	(-)	0.4 %
		24	(-)	1.17 %
		48	(-)	1.51 %
NiO[b]	NP	0	0.64 %	0.35 %
		2	(-)	(-)
		24	0.81 %	1.16 %
		48	0.94 %	1.81 %
	MP	0	(-)	0.018 %
		2	(-)	(-)
		24	(-)	0.052 %
		48	(-)	0.067 %
Al_2O_3[a]	NP	0	1 %	0.93 %
		2	(-)	(-)
		24	1.25 %	0.75 %
		48	1.11 %	0.73 %
	MP	0	(-)	0.02 %
		2	(-)	(-)
		24	(-)	0.038 %
		48	(-)	0.021 %
TiO_2[b]	NP	0	0.118 %	0.014 %
		2	0.128 %	0.077 %
		24	0.17 %	0.045 %
		48	(-)	(-)
	MP	0	(-)	(-)
		2	(-)	0.0006 %
		24	(-)	0.0005 %
		48	(-)	(-)

[a] Data from Avramescu et al., 2023 [32]. [b] Data from Avramescu et al., 2020 [31].

Table S2. The top ten most commonly enriched IPA canonical pathways following 2-48 h of exposure to each separate metal oxide form. MONP: metal oxide nanoparticle. MOMP: metal oxide microparticle. MeCl: metal chloride.

Form	Pathway	Total Number of Samples Where Pathway is Enriched
MONP	HIF1α Signaling	17/26
	Pulmonary Fibrosis Idiopathic Signaling Pathway	16/26
	ID1 Signaling Pathway	16/26
	Aryl Hydrocarbon Receptor Signaling	15/26
	Hepatic Fibrosis Signaling Pathway	15/26
	IL-10 Signaling	15/26
	Hepatic Fibrosis / Hepatic Stellate Cell Activation	14/26
	Role of Macrophages, Fibroblasts and Endothelial Cells in Rheumatoid Arthritis	14/26
	Role Of Chondrocytes In Rheumatoid Arthritis Signaling Pathway	14/26
	Role of Osteoblasts, Osteoclasts and Chondrocytes in Rheumatoid Arthritis	14/26
	Role Of Osteoblasts In Rheumatoid Arthritis Signaling Pathway	14/26
	Osteoarthritis Pathway	14/26
	CLEAR Signaling Pathway	14/26
MOMP	LPS/IL-1 Mediated Inhibition of RXR Function	11/17
	HIF1α Signaling	8/17
	Role of Macrophages, Fibroblasts and Endothelial Cells in Rheumatoid Arthritis	8/17
	Agranulocyte Adhesion and Diapedesis	8/17
	Atherosclerosis Signaling	8/17
	Granulocyte Adhesion and Diapedesis	8/17
	Axonal Guidance Signaling	6/17
	Osteoarthritis Pathway	6/17
	NRF2-mediated Oxidative Stress Response	6/17
	Sirtuin Signaling Pathway	6/17
	Pathogen Induced Cytokine Storm Signaling Pathway	6/17
	Antioxidant Action of Vitamin C	6/17
	Role Of Osteoblasts In Rheumatoid Arthritis Signaling Pathway	6/17
	Acute Phase Response Signaling	6/17
	Hepatic Fibrosis / Hepatic Stellate Cell Activation	6/17
	LXR/RXR Activation	6/17
MeCl	Pulmonary Fibrosis Idiopathic Signaling Pathway	11/15
	Tumor Microenvironment Pathway	11/15
	HIF1α Signaling	10/15
	Aryl Hydrocarbon Receptor Signaling	10/15
	Wound Healing Signaling Pathway	9/15
	Hepatic Fibrosis / Hepatic Stellate Cell Activation	9/15
	p53 Signaling	9/15
	Ferroptosis Signaling Pathway	9/15
	Role of Macrophages, Fibroblasts and Endothelial Cells in Rheumatoid Arthritis	9/15
	ID1 Signaling Pathway	9/15
	Hepatic Fibrosis Signaling Pathway	9/15

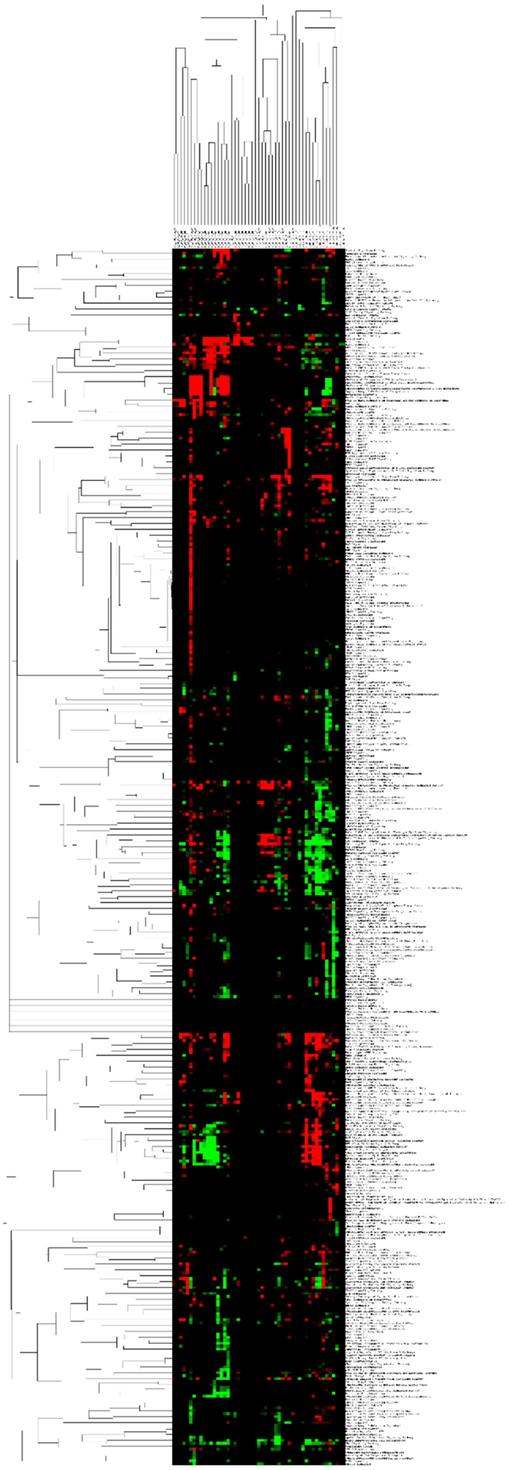

Figure S5. Hierarchical clustering of exposure groups (x-axis) and significantly enriched IPA canonical pathways (y-axis) using z-score. Green: negative z-score (min: -2), red: positive z-score (max: 2).

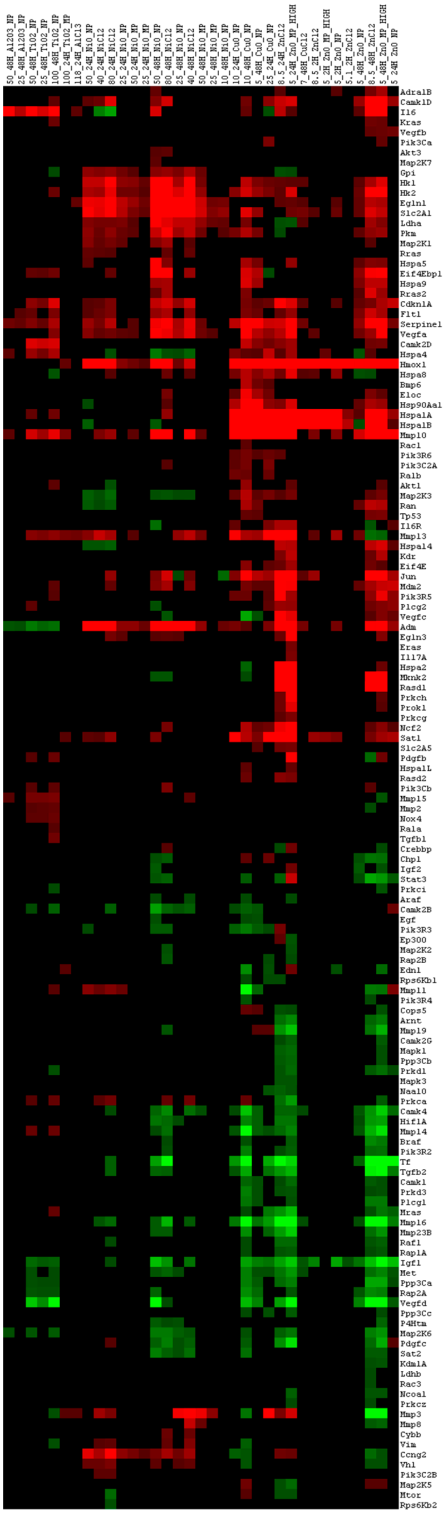

Figure S6. Heatmap depicting differential gene expression in the 'HIF-1α Signaling' IPA canonical pathway. Red: positive fold change (max = 5). Green: negative fold change (max = -5). A gene was considered differentially expressed if the |FC| > 1.5, with an adjusted $p < 0.05$. The 'HIF-1α Signaling' pathway was considered significantly enriched if the -log(pvalue) > 1.3, with at least 3 contained DEGs.

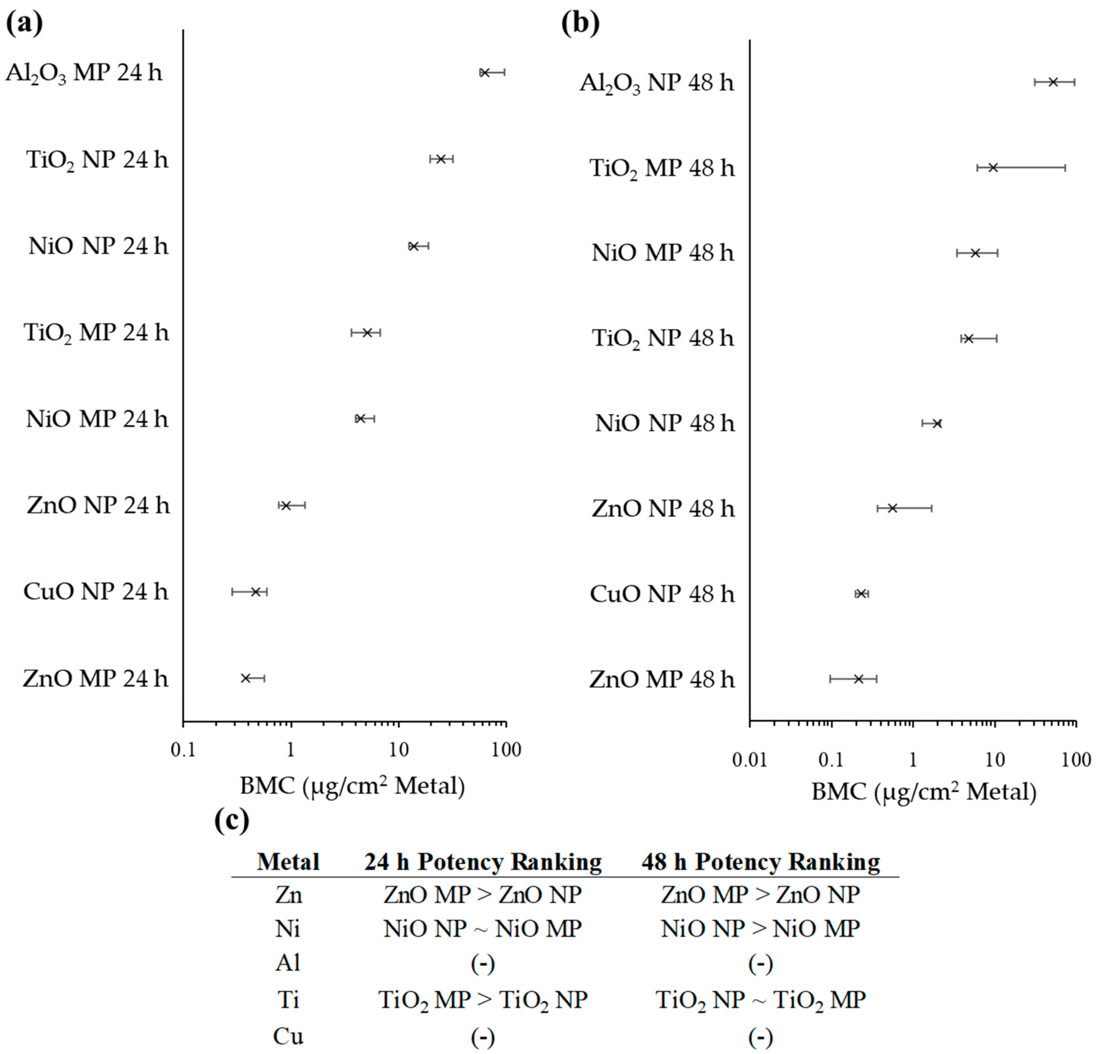

(c)

Metal	24 h Potency Ranking	48 h Potency Ranking
Zn	ZnO MP > ZnO NP	ZnO MP > ZnO NP
Ni	NiO NP ~ NiO MP	NiO NP > NiO MP
Al	(-)	(-)
Ti	TiO_2 MP > TiO_2 NP	TiO_2 NP ~ TiO_2 MP
Cu	(-)	(-)

Figure S7. BMDS based BMC modelling of decreases in viable cell density following (a) 24 h and (b) 48 h exposure to MONPs and MOMPs, with (c) differences in potency for each metal variety. The concentration is expressed in terms of specific surface area, using the average estimate for MOMPs. Benchmark response: 0.5 (Hybrid extra risk). The 'x' indicates the BMC. Left and right bars indicate BMCL and BMCU values respectively. NP: nanoparticle. MP: microparticle.

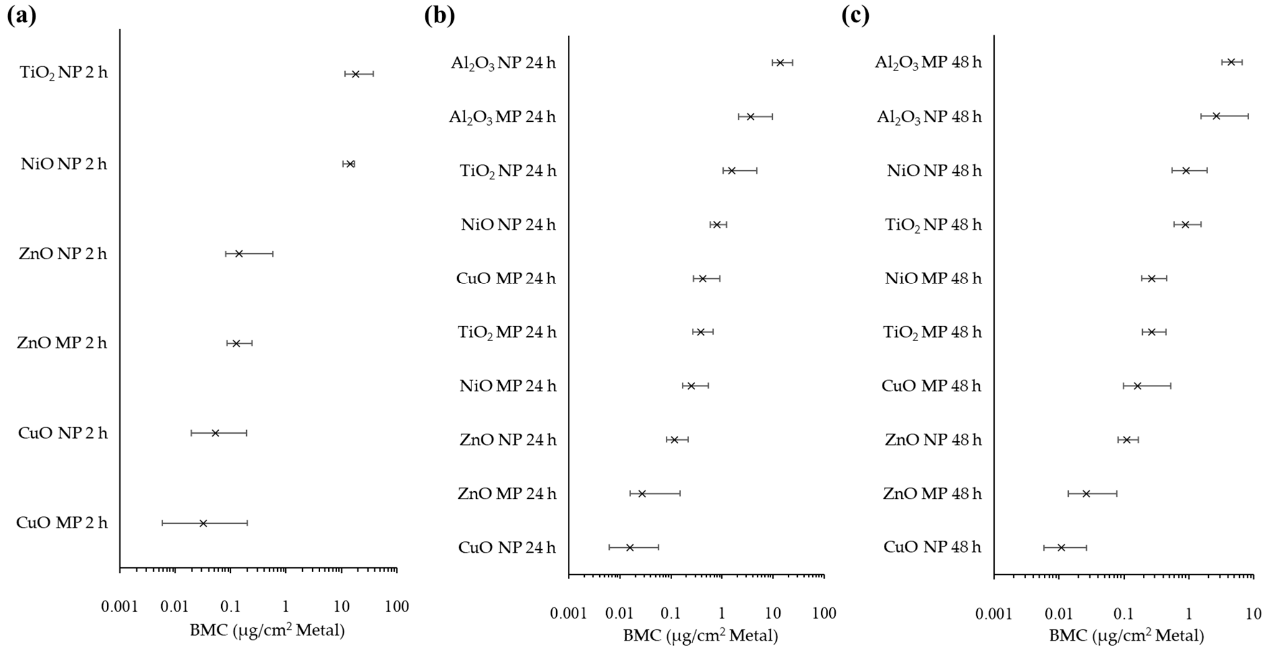

(d)

Metal	2 h Potency Ranking	24 h Potency Ranking	48 h Potency Ranking
Zn	ZnO NP ~ ZnO MP	ZnO NP ~ ZnO MP	ZnO MP > ZnO NP
Ni	(-)	NiO MP > NiO NP	NiO MP > NiO NP
Al	(-)	Al_2O_3 MP ~ Al_2O_3 NP	Al_2O_3 MP ~ Al_2O_3 NP
Ti	(-)	TiO_2 MP > TiO_2 NP	TiO_2 MP > TiO_2 NP
Cu	CuO NP ~ CuO MP	CuO NP > CuO MP	CuO NP > CuO MP

Figure S8. The 25th ranked gene tPOD determined through BMDExpress2 BMC modelling for (a) 2 h, (b) 24 h, and (c) 48 h MONP and MOMP exposures, with d) differences in potency for each metal variety. The concentration is expressed in terms of specific surface area, using the average estimate for MOMPs. Benchmark response: 1 (Standard deviation). The 'x' indicates the BMC. Left and right bars indicate BMCL and BMCU values respectively. NP: nanoparticle. MP: microparticle.
